# Dietary Soy Isoflavones Ameliorate Muscle Quality in High-Fat Diet-Fed Rice Field Eels (*Monopterus albus*) by Modulating Myogenesis, Collagen Synthesis, and Antioxidant Capacity

**DOI:** 10.3390/antiox14101195

**Published:** 2025-10-01

**Authors:** Kai Xie, Quan Li, Shuang Zheng, Huahong Wei, Tao Zhou, Yi Hu, Junzhi Zhang

**Affiliations:** 1 College of Fisheries, Hunan Agricultural University, Changsha 410128, China; xk971006@stu.hunau.edu.cn (K.X.); liquan4016@stu.hunau.edu.cn (Q.L.); zhengs@stu.hunau.edu.cn (S.Z.); whh0420@stu.hunau.edu.cn (H.W.); zhoutao0425@stu.hunau.edu.cn (T.Z.); 2 Hunan Engineering Technology Research Center of Featured Aquatic Resources Utilization, Hunan Agricultural University, Changsha 410128, China

**Keywords:** *Monopterus albus*, soy isoflavones, high-fat diet, fatty-acid composition, myogenesis, collagen synthesis, antioxidant capacity

## Abstract

High-fat diets are increasingly used to improve feed efficiency in aquaculture but may deteriorate fillet quality and health; soy isoflavones, plant-derived polyphenols, have emerged as promising modulators of muscle growth, antioxidant defense, and lipid metabolism in fish. This study investigated the effects of dietary soy isoflavone supplementation on myogenesis, collagen synthesis, fatty-acid composition, and antioxidant capacity in muscle of *Monopterus albus* fed a high-fat diet. Fish were assigned to four diets: control (CON, 6.16% crude fat), high-fat without soy isoflavones (HSIF0, 11.98% crude fat), and high-fat with 50 mg/kg (HSIF50) or 100 mg/kg (HSIF100) soy isoflavones. HSIF0 significantly elevated whole-body/muscle lipids, reduced ΣSFA/ΣMUFA/Σn-3/Σn-6 ratios (*p* < 0.05), increased Σn-6 (*p* < 0.05), impaired water-holding capacity/texture (higher losses, lower hardness/cohesiveness/gumminess/chewiness/resilience) (*p* < 0.05), induced loosely arranged myofibers with enlarged inter-fiber spaces, downregulated myogenesis (upregulated mstn; downregulated myod/tcap/mrf4/mrf5) and collagen genes (ets1/sp1/p4ha1) (*p* < 0.05), decreased collagen/hydroxyproline (*p* < 0.05), and weakened antioxidants (higher MDA/H_2_O_2_; lower T-AOC/GSH; downregulated nrf2/sod/cat/gpx1/gpx8) (*p* < 0.05). HSIF50 reversed these effects, enhancing ΣPUFA/Σn-3/EPA+DHA (*p* < 0.05), restoring structure/gene expression (*p* < 0.05), and boosting antioxidants (*p* < 0.05). In contrast, HSIF100 partially diminished benefits, indicating dose-dependency. Overall, 50 mg/kg soy isoflavones optimally mitigated high-fat-induced muscle quality decline via lipid remodeling, structural improvement, collagen promotion, and antioxidant enhancement.

## 1. Introduction

In aquaculture, high-fat diets have gained prominence due to their numerous advantages in enhancing fish production efficiency. These diets provide a concentrated energy source that is cost-effective, as lipids are generally cheaper than proteins, allowing for protein-sparing effects where dietary protein can be reduced without compromising growth [1]. However, high-fat diets promote ectopic lipid deposition in muscle and viscera, elevate malondialdehyde (MDA; lipid-peroxidation marker), and suppress antioxidant enzymes such as superoxide dismutase (SOD) and glutathione peroxidase (GPx). These changes increase reactive oxygen species (ROS), damaging cells, and triggering inflammation. As a result, flesh quality deteriorates—firmness declines, drip loss rises, and fatty-acid profiles shift toward pro-inflammatory n-6 polyunsaturated fatty acids (PUFAs) [2]. Studies indicate that hypoxia in Nile tilapia (*Oreochromis niloticus*) reduces growth rate, alters muscle proximate composition and flesh quality, increases lipid accumulation, and compromises texture parameters such as hardness and chewiness [3]. In grass carp (*Ctenopharyngodon idella*), high fat levels altered muscle growth traits, decreasing collagen synthesis and myofiber density, which correlated with softer textures and reduced water-holding capacity [4]. Similarly, high-fat diets in rainbow trout (*Oncorhynchus mykiss*) resulted in reduced myostatin-1 gene activity and modified lipid profiles in muscle tissue, which compromised muscle fiber development and oxidative defenses, consequently causing withered or less elastic muscle [5]. In yellow catfish (*Pelteobagrus fulvidraco*), high-fat diets induced oxidative stress, reducing fatty acid oxidation and collagen levels, further degrading muscle quality [6]. Collectively, these lines of evidence underscore an urgent need to rationalize high-fat feeding strategies in aquaculture—optimizing lipid level and source and leveraging functional additives—to sustain growth while safeguarding flesh quality and fish health. Yet, the underlying mechanisms by which high-fat diets impair muscle quality and the nutritional interventions that can effectively mitigate these effects remain insufficiently understood.

Soy isoflavones (SIFs), primarily genistein and daidzein, are bioactive phytoestrogens abundant in soybeans and soy-based feeds, known for their estrogen-mimicking properties and potential health-modulating effects [7]. They are recognized as potent antioxidants, capable of scavenging free radicals, inhibiting lipid peroxidation, and enhancing the activity of endogenous antioxidant enzymes [8]. In livestock such as broilers and pigs, SIFs enhance meat quality by directly improving antioxidative properties, which in turn helps stabilize protein matrices and mitigate the oxidative stress that would otherwise compromise meat quality. They also boost redness, water-holding capacity, and lean muscle growth [9,10]. In aquatic animals, SIFs offer similar benefits; for instance, in turbot (*Scophthalmus maximus*) [11] and juvenile golden pompano (*Trachinotus ovatus*) [12], SIFs supplemented in feeds enhance antioxidant defenses, immune responses, and fat metabolism, whereas research on grass carp indicates improvements in flesh physical properties like tenderness and water-holding capacity through enhanced collagen deposition and flavor quality [13]. Soy isoflavones activate nuclear factor erythroid 2–related factor 2 (Nrf2)-dependent antioxidant defenses and improve redox status; as phytoestrogens they also interact with estrogen receptors, which can affect muscle signaling and extracellular-matrix homeostasis in a context- and dose-dependent manner [8]. However, excessive SIF addition can elicit adverse outcomes, particularly in fish, where high doses may disrupt endocrine functions, induce pro-oxidant, and compromise growth and quality [14]. For example, in rice field eels (*Monopterus albus*), levels above 2.5 g/kg depressed growth responses, feed efficiency ratios and triggered inflammation [15]. High SIF levels in Chinese mitten crabs (*Eriocheir sinensis*) also compromised oxidative defenses and innate immune functions [16], while in rainbow trout, they potentially induced stress responses like elevated cortisol, leading to diminished muscle quality and reproductive issues [17]. While SIFs are known to improve muscle quality in terrestrial animals and some fish, their role in rice field eel muscle quality under high-fat diets has not been studied. The dose-dependent effects of SIFs (beneficial or harmful) are poorly understood in aquaculture species. Therefore, the aim of this study was to investigate the effects of soy isoflavones on muscle quality, antioxidant capacity and collagen synthesis in rice field eels fed a high-fat diet and to determine the optimal inclusion levels.

## 2. Materials and Methods

### 2.1. Ethical Statement

All experimental procedures were conducted in strict accordance with the guidelines for the care and use of laboratory animals established by the Ministry of Science and Technology of the People’s Republic of China (Guidelines for the Welfare of Laboratory Animals, 2006) and complied with international standards, including the ARRIVE guidelines for reporting animal research. This study was approved by the Committee of Laboratory Animal Management and Animal Wel-fare of Hunan Agricultural University (Changsha, China), and all experimental procedures con-formed to the guidelines of the Ethical Committee of Hunan Agricultural University (approval number: 2025121, approved on 8 April 2025).

### 2.2. Experimental Diets

The test feeds were prepared utilizing fish meal, soy protein concentrate and poultry by-product meal as main protein components, along with fish oil serving as the fat supplier. Every ingredient for the feeds came from Zhangjiajie Xinrui Feeds Co., Ltd. (Zhangjiajie, China). Soy isoflavones were provided as a commercial preparation (≥98%, purchased from Chengdu Yuancheng Technology Co., Ltd., Chengdu, China). Four distinct feeds were designed: a control diet (CON) containing 6.16% crude fat, a high-fat diet (containing 11.98% crude fat) without soy isoflavones (HSIF0), plus two high-fat diets enriched with either 50 mg/kg soy isoflavones (HSIF50) or 100 mg/kg soy isoflavones (HSIF100). Prior to formulating the feeds, each raw material was milled into a fine consistency and blended progressively in suitable ratios to achieve uniform distribution. Soy isoflavones were first premixed with a small portion of the basal powder (1:10, *w*/*w*) to ensure uniform dispersion, and then the premix was homogenized with the remaining ingredients using a V-type mixer for 5 min before the addition of fish oil. Fish oil was incorporated directly into the blend to maintain even texture. The resulting diets were air-dried and kept in a cool, dry place prior to feeding. Detailed diet formulations are listed in Table 1.

### 2.3. Feeding Experiment and Sample Collection

The *M. albus* utilized in this experiment were procured from Xihu Farm, situated in Changde, China, and belonged to a variety distinguished by deep yellow coloration with large spots. A total of 800 fish, with an average initial body weight of 26 g, were randomly allocated to 16 cages measuring 2.0 m × 1.5 m × 1.5 m. After a two-week acclimation period and 24 h of fasting, 50 eels were placed in each cage, and each treatment group had 4 replicates. In this study, experimental units (such as tanks or animals) were allocated to control and treatment groups using randomisation. The randomisation sequence was generated using the random number function in Excel. The acclimation procedure followed the methods from our previous work [18]. Throughout the 8-week trial period, the fish received manual feeding once per day from 17:00 to 18:30, with rations set at 3–5% of their body mass. Water quality was maintained throughout the experiment, with pH ranging from 7.1 to 7.5, dissolved oxygen above 6.5 mg/L, an average temperature of 29.0 °C, and ammonia levels below 0.5 mg/L. In cases where an individual eel exhibited signs of severe distress, such as continuous loss of appetite and inactivity on the aquatic plants for several consecutive days, it would be humanely euthanized to prevent further suffering.

After fasting for 24 h, seven eels were arbitrarily chosen from every enclosure and sedated using eugenol, followed by rapid euthanasia. Dorsal muscle was dissected from both sides of the spine. For three fish, approximately 1–2 cm^3^ of dorsal muscle was collected for texture profile analysis (stored in 2 mL tubes at 4 °C) and histological examination (fixed in 4% paraformaldehyde in 15 mL tubes). For one fish, ~6 g of dorsal muscle was collected for water loss determination. For another fish, ~2.0 g of dorsal muscle was placed in RNase-free 1.5 mL tubes, rapidly snap-frozen using liquid nitrogen, then preserved at −80 °C until further biochemical and gene expression evaluations. For the remaining two fish, ~10 g of dorsal muscle was placed in sealed polyethylene bags and stored at −20 °C for proximate composition and fatty acid profile analysis. The lab technician performing the outcome assessments was blinded to the group assignments. A schematic representation of the experimental design is provided in Figure S1.

### 2.4. Analytical Methods

#### 2.4.1. Proximate Composition of Feed, Muscle and Whole Fish

Proximate composition of feed, muscle, and whole-body samples was analyzed according to AOAC official methods [19]. Moisture levels were quantified by oven-drying specimens at 105 °C to reach stable mass. Lipids were isolated via Soxhlet extraction with diethyl ether solvent. Mineral content was measured by incinerating materials in a muffle oven at 550 °C until mass remained unchanged.

#### 2.4.2. Muscle Fatty Acid Composition

Approximately 2.0 g of dorsal muscle was freeze-dried and ground into a fine powder. A 100 mg portion of the sample was extracted with 2 mL of chloroform–methanol (2:1, *v*/*v*). Following centrifugation, the upper layer was harvested, and the extracted lipids underwent methylation using 2 mL of methanol with 1% sulfuric acid and 0.01% butylated hydroxytoluene (BHT) at 80 °C for 2 h, yielding fatty acid methyl esters (FAMEs). The fatty acid profiles were examined via gas chromatography-mass spectrometry (GC-MS; model 7890B-5977A, Agilent, Santa Clara, CA, USA) fitted with an HP-5MS capillary column (30 m × 0.25 mm i.d. × 0.25 μm film thickness). Helium served as the carrier gas, flowing at 1 mL/min, with a 1 μL sample injection volume. The column temperature program started at 50 °C for 2 min, ramped up to 220 °C at a rate of 10 °C/min, and maintained for 15 min. Fatty acid contents (mg/g dry weight) were quantified using FAME standards (Supelco, Bellefonte, PA, USA) as external references, and bar charts were subsequently generated from the relative abundance of each fatty acid (percentage of total identified fatty acids).

#### 2.4.3. Muscle Water-Holding Capacity

Cooking loss, liquid loss, and water loss of dorsal muscle were determined according to Honikel (1998) with modifications [20]. Sample weights were measured to the nearest 0.0001 g using an electronic analytical balance (FA1004B, Shanghai Precision & Scientific Instrument Co., Ltd., Shanghai, China).

Approximately 2.0 g of dorsal muscle (recorded as W1) was sealed in a polyethylene bag, immersed in in a water bath maintained at 80 °C for 20 min, subsequently cooled to ambient temperature, and patted dry. The post-cooking weight (W2) was then measured. The percentage of cooking loss was determined by the equation: [(W1 − W2)/W1] × 100%.

Approximately 2.0 g of dorsal muscle tissue (designated W1) was enclosed in a plastic bag and refrigerated at 4 °C for 48 h. Accumulated fluid was discarded, and the tissue was reweighed (W2). The percentage of liquid loss was determined by the equation: [(W1 − W2)/W1] × 100%.

Approximately 2.0 g of dorsal muscle tissue was precisely measured (denoted as W1), positioned between two sheets of filter paper, and compressed with a 35 kg load for 5 min. Post-compression, the tissue was reweighed (denoted as W2). The percentage of water loss was determined using the equation: [(W1 − W2)/W1] × 100%.

#### 2.4.4. Muscle Textural Properties

Muscle textural properties, including hardness, springiness, cohesiveness, gumminess, chewiness, and resilience, were measured using a texture profile analysis (TPA) method with a texture analyzer (TA.XT Plus, Stable Micro Systems, Godalming, Surrey, UK) equipped with a P/36R flat cylindrical probe. Raw dorsal muscle samples (1–2 cm^3^) were compressed twice to 50% of their original height at a constant speed of 1 mm/s, with a 5 s interval between compressions. Data were analyzed using the instrument’s software (Exponent software, version 2.21.3) to calculate each texture parameter.

#### 2.4.5. Muscle Histomorphology

Muscle tissue samples were fixed in 4% paraformaldehyde and processed following dehydration, clearing, embedding in paraffin, sectioning, and deparaffinization. After sectioning, the slides were stained with H&E staining. After section preparation, the slides were scanned and observed using an Eclipse Ci-L camera (Nikon, Tokyo, Japan) and a KF-FL-020 Digital Whole-Slide Pathology Scanner (Ningbo Jiangfeng Bioinformatics Technology Co., Ltd., Zhejiang, China).

The other muscle tissue samples were embedded in OCT, snap-frozen, and stored at −80 °C. Cryosections (8–10 μm) were cut at −20 °C, air-dried, fixed in 4% PFA for 10 min, rinsed in PBS, equilibrated in 60% isopropanol for 1 min, followed by incubation in a newly prepared Oil Red O solution (made by diluting 0.5% Oil Red O isopropanol stock in a 3:2 ratio with water, followed by filtration) for 10–15 min under ambient conditions. Slides underwent a short differentiation step in 60% isopropanol (approximately 30 s), followed by a water rinse and hematoxylin counterstaining (30–60 s), and mounted with glycerol gelatin. Images were acquired with the same microscope/scanner as above.

#### 2.4.6. Muscle Collagen Content

Muscle collagen content was determined based on hydroxyproline measurement. The hydroxyproline content was determined based on its reaction with an oxidizing agent to produce oxidative products, which subsequently react with pdimethylaminobenzaldehyde to yield a purplish-red color. The color intensity was used to calculate the hydroxyproline content, using a commercial assay kit (Nanjing Jiancheng Bioengineering Institute, Nanjing, China). Collagen content was calculated assuming hydroxyproline accounts for 13.4% of the total collagen.

#### 2.4.7. Muscle Antioxidant Indices

Malondialdehyde (MDA) content was measured by reacting with thiobarbituric acid to form a pink complex, which was then measured at 532 nm. Superoxide dismutase (SOD) activity was assessed by monitoring the reduction of nitrogen blue tetrazole at 450 nm. Total antioxidant capacity (T-AOC) was measured using the ferric reducing ability of plasma (FRAP) method, in which antioxidants reduce the Fe^3+^-TPTZ complex to Fe^2+^-TPTZ, producing a blue color with a maximum absorbance at 593 nm. The content of glutathione (GSH) was quantified by the reaction with 2-nitrobenzoic acid, producing yellow 2-nitro-5-mercaptobenzoic acid, with a characteristic absorption peak at 405 nm. Hydrogen peroxide (H_2_O_2_) content was quantified based on the reaction of H_2_O_2_ with molybdic acid to form a yellow complex, which was measured at 405 nm.

#### 2.4.8. Real-Time Polymerase Chain Reaction

Total RNA was isolated from muscle tissues using Trizol reagent (Monad Biotech Co., Suzhou, China). RNA quality was assessed, and reverse transcription was performed with MonScript™ RTIII Super Mix (Yeasen Biotechnology, Shanghai, China). Quantitative PCR (qPCR) was conducted using SYBR^®^ Green qPCR Mix (Monad Biotech Co., Suzhou, China), the cycling parameters were 95 °C for 3 min; 40 cycles of 95 °C for 10 s and 60 °C for 15 s; followed by a melt curve from 65 to 95 °C, 0.5 °C increments. Gene expression associated with muscle myogenesis, collagen metabolism and antioxidant were quantified, with primer sequences provided in Table 2.

#### 2.4.9. Statistical Analysis

Data processing was carried out in Microsoft Excel 2019, while statistical evaluations were performed using R version 4.3.3. The experimental unit was the cage (*n* = 4 per treatment). For variables measured on multiple fish per cage, individual values were averaged within each cage prior to analysis. Data normality was evaluated with the Shapiro–Wilk test, and homogeneity of variance was assessed using Levene’s test. When assumptions were met, one-way ANOVA followed by Tukey’s HSD test was applied (*p* < 0.05). For non-normally distributed datasets, the Kruskal–Wallis test followed by pairwise comparisons with Bonferroni adjustment was used.

## 3. Results

### 3.1. Proximate Composition, Muscle Oil Red O-Stained Sections, and Fatty Acid Profile

**Figure 1:**
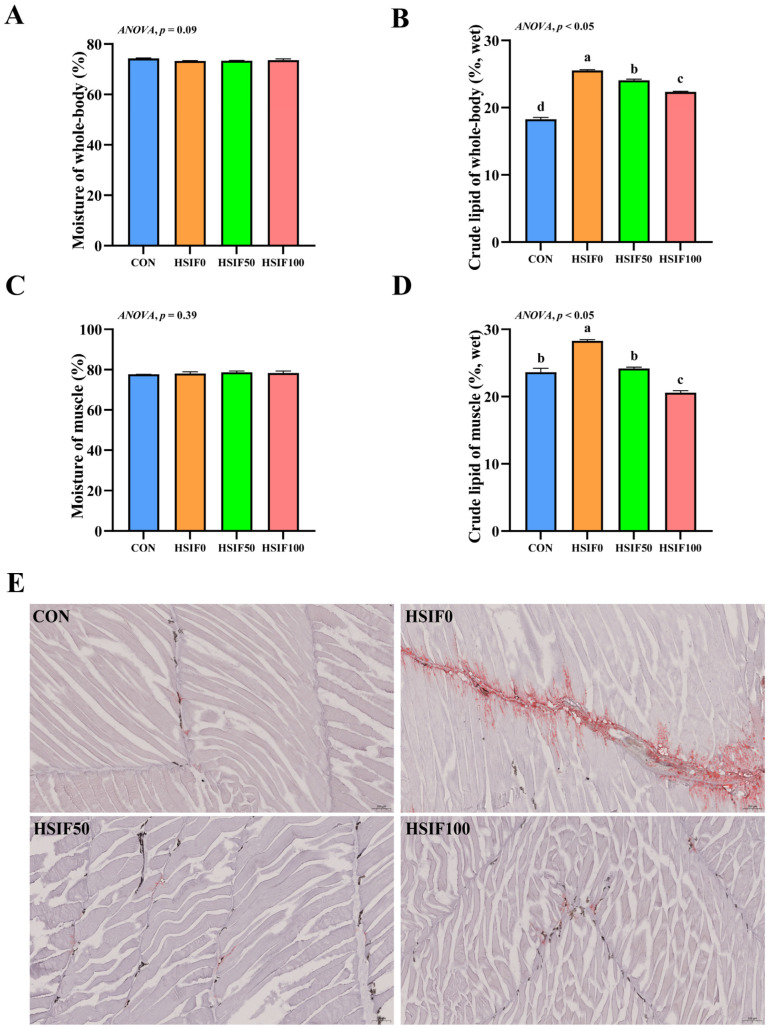
Effects of dietary treatments on proximate composition, muscle Oil red O-stained sections in *M. albus*. Notes: (**A**) Moisture of whole-body (*n* = 4); (**B**) crude lipid of whole-body (*n* = 4); (**C**) Moisture of muscle (*n* = 4); (**D**) crude lipid of muscle (*n* = 4); (**E**) The image shows Oil Red O-stained sections (150×). CON: control diet (6.16% crude fat); HSIF0: high-fat diet (11.98% crude fat); HSIF50: HSIF0 supplemented with 50 mg/kg soy isoflavones; HSIF100: HSIF0 supplemented with 100 mg/kg soy isoflavones. Bars represent mean ± standard error (SE). Different superscript letters denote significant differences among groups (*p* < 0.05). CON: control diet (6.16% crude fat); HSIF0: high-fat diet (11.98% crude fat); HSIF50: HSIF0 supplemented with 50 mg/kg soy isoflavones; HSIF100: HSIF0 supplemented with 100 mg/kg soy isoflavones.

In this study, no significant differences in muscle moisture content were observed among the treatment groups (*p* > 0.05). Compared with the CON group, whole-body and muscle crude lipid contents were significantly higher in the HSIF0 group (*p* < 0.05). Supplementation of the high-fat diet with 50 or 100 mg/kg soy isoflavones significantly reduced whole-body and muscle crude lipid contents compared with HSIF0 (*p* < 0.05), with the HSIF100 group showing a further decrease relative to HSIF50 (*p* < 0.05) (Figure 1A–D).

As shown in Figure 1E, the CON group exhibited almost no Oil Red O–positive staining, indicating minimal intramuscular lipid. In contrast, the HSIF0 group showed extensive red staining distributed along myosepta and between fibers, reflecting marked lipid droplet accumulation. Supplementation with 50 mg/kg and 100 mg/kg soy isoflavones markedly attenuated staining intensity, with only sparse punctate droplets.

**Figure 2:**
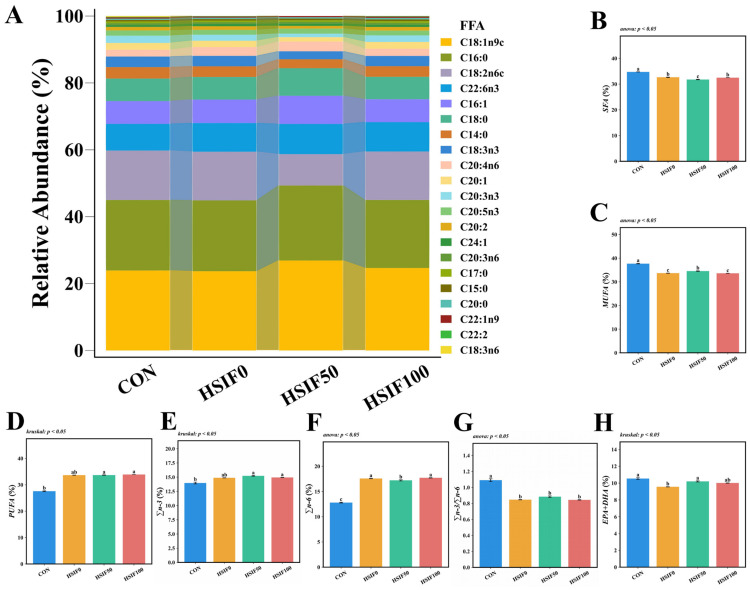
Effects of dietary treatments on muscle fatty acid composition in *M. albus* (*n* = 4). Notes: (**A**) interactive ribbon bar chart; (**B**) total saturated fatty acids (ΣSFA); (**C**) total monounsaturated fatty acids (ΣMUFA); (**D**) total polyunsaturated fatty acids (ΣPUFA); (**E**) total n-3 polyunsaturated fatty acids (Σn-3); (**F**) total n-6 polyunsaturated fatty acids (Σn-6); (**G**) ratio of total n-3 to total n-6 polyunsaturated fatty acids (Σn-3/Σn-6). (**H**) eicosapentaenoic acid plus docosahexaenoic acid (EPA + DHA) Bars represent mean ± standard error (SE). Different superscript letters denote significant differences among groups (*p* < 0.05). CON: control diet (6.16% crude fat); HSIF0: high-fat diet (11.98% crude fat); HSIF50: HSIF0 supplemented with 50 mg/kg soy isoflavones; HSIF100: HSIF0 supplemented with 100 mg/kg soy isoflavones.

As shown in Figure 2, the five most abundant fatty acids in *M. albus* muscle were C18:1n9c, C16:0, C18:2n6c, C22:6n3, and C16:1. Compared with the CON group, the HSIF0, HSIF50, and HSIF100 groups showed significantly lower ΣSFA, ΣMUFA, and Σn-3/Σn-6 ratios, but higher Σn-6 proportions (*p* < 0.05). In contrast, ΣPUFA and Σn-3 proportions were significantly higher in the HSIF50 and HSIF100 groups than in the CON group (*p* < 0.05). Furthermore, compared with HSIF0, the HSIF50 group exhibited significantly lower ΣSFA and Σn-6 proportions, but higher ΣMUFA and EPA + DHA proportions (*p* < 0.05).

### 3.2. Muscle Water-Holding Capacity and Textural Properties

**Figure 3:**
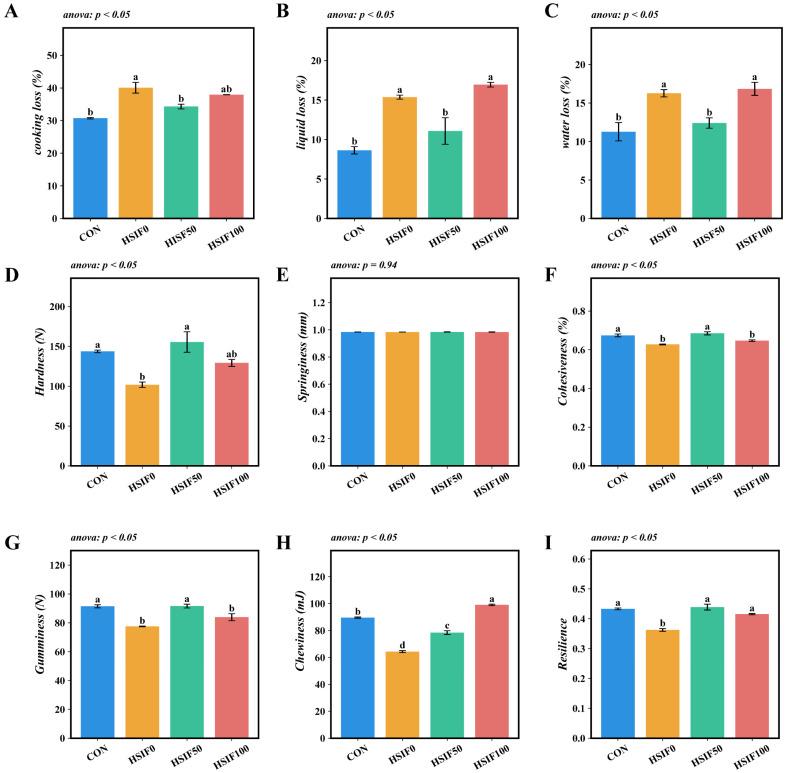
Effects of dietary treatments on muscle water-holding capacity and textural properties in *M. albus* (*n* = 4). Notes: (**A**) cooking loss of muscle; (**B**) liquid loss of muscle; (**C**) water loss of muscle; (**D**) hardness (**E**) springiness; (**F**) cohesiveness; (**G**) gumminess; (**H**) chewiness; (**I**) resilience. CON: control diet (6.16% crude fat); HSIF0: high-fat diet (11.98% crude fat); HSIF50: HSIF0 supplemented with 50 mg/kg soy isoflavones; HSIF100: HSIF0 supplemented with 100 mg/kg soy isoflavones. Bars represent mean ± standard error (SE). Different superscript letters denote significant differences among groups (*p* < 0.05). CON: control diet (6.16% crude fat); HSIF0: high-fat diet (11.98% crude fat); HSIF50: HSIF0 supplemented with 50 mg/kg soy isoflavones; HSIF100: HSIF0 supplemented with 100 mg/kg soy isoflavones.

Compared with the CON group, the HSIF0 group exhibited significantly higher cooking loss, liquid loss, and water loss (*p* < 0.05). Compared with the HSIF0 group, supplementation of the high-fat diet with 50 mg/kg soy isoflavones significantly reduced cooking loss, liquid loss, and water loss (*p* < 0.05). Furthermore, the HSIF100 group showed significantly higher liquid loss and water loss than the HSIF50 group (*p* < 0.05) (Figure 3A–C).

No significant differences in muscle springiness were observed among the dietary treatments (*p* > 0.05). Compared with the CON group, the HSIF0 group showed significantly lower hardness, cohesiveness, gumminess, chewiness, and resilience (*p* < 0.05). Supplementation of the high-fat diet with 50 mg/kg soy isoflavones (HSIF50) significantly increased these five textural parameters relative to the HSIF0 group (*p* < 0.05). However, as the level of soy isoflavone supplementation increased, cohesiveness, gumminess, and chewiness decreased significantly (*p* < 0.05) (Figure 3D–I).

### 3.3. Muscle Histomorphology and Expression of Myogenesis-Related Genes

**Figure 4:**
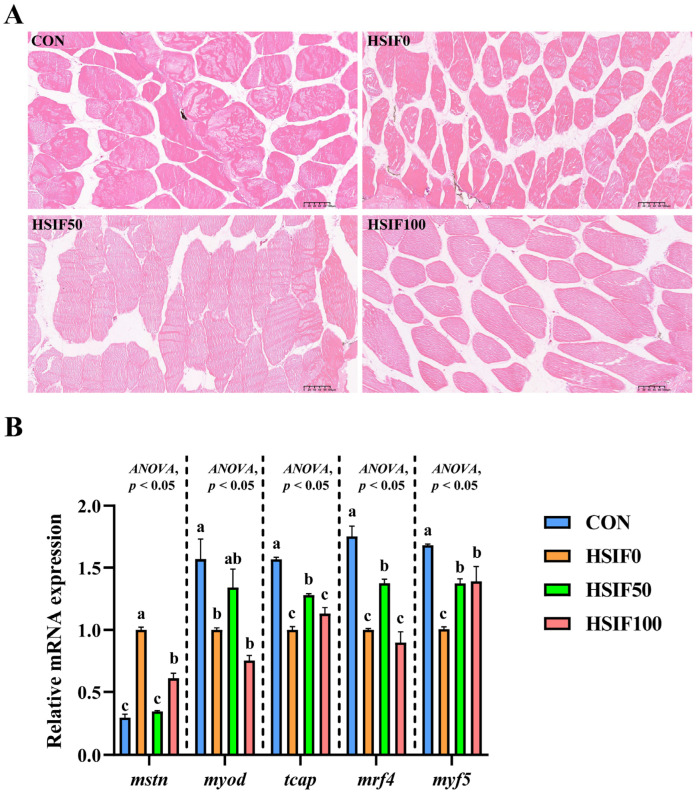
Effects of dietary treatments on muscle histomorphology and expression of myogenesis-related genes in *M. albus*. Notes: (**A**) The image shows H&E-stained sections (150×). (**B**) *n* = 4; mstn—Myostatin; myod—Myogenic differentiation; tcap—Telethonin; mrf4—Myogenic regulatory factor 4; mrf5—Myogenic regulatory factor 5. rpl17 was the internal reference gene. Bars represent mean ± standard error (SE). Different superscript letters denote significant differences among groups (*p* < 0.05). CON: control diet (6.16% crude fat); HSIF0: high-fat diet (11.98% crude fat); HSIF50: HSIF0 supplemented with 50 mg/kg soy isoflavones; HSIF100: HSIF0 supplemented with 100 mg/kg soy isoflavones.

As shown in Figure 4A, muscle fibers in the CON group were arranged compactly with regular morphology and minimal inter-fiber spaces. In the HSIF0 group, fibers exhibited loose arrangement, irregular shapes, and enlarged inter-fiber spaces, with partial structural disruption. Supplementation of the high-fat diet with 50 mg/kg soy isoflavones (HSIF50) resulted in tightly packed fibers, more uniform morphology, and reduced inter-fiber gaps compared with the HSIF0 group. However, in the HSIF100 group, although the fibers remained generally intact, the inter-fiber spaces were larger than in the HSIF50 group, indicating a partial decline in muscle structural integrity at higher supplementation levels.

As shown in Figure 4B, compared with the CON group, the HSIF0 group exhibited a significant upregulation of mstn expression and a significant downregulation of myod, tcap, mrf4, and mrf5 expression (*p* < 0.05). Compared with the HSIF0 group, supplementation of the high-fat diet with 50 mg/kg soy isoflavones significantly downregulated mstn expression and significantly upregulated tcap, mrf4, and mrf5 expression (*p* < 0.05). Furthermore, in the HSIF100 group, mstn expression was significantly upregulated, whereas tcap and mrf4 expression were significantly downregulated compared with the HSIF50 group (*p* < 0.05).

### 3.4. Muscle Collagen Content and Expression of Collagen Metabolism-Related Genes

**Figure 5:**
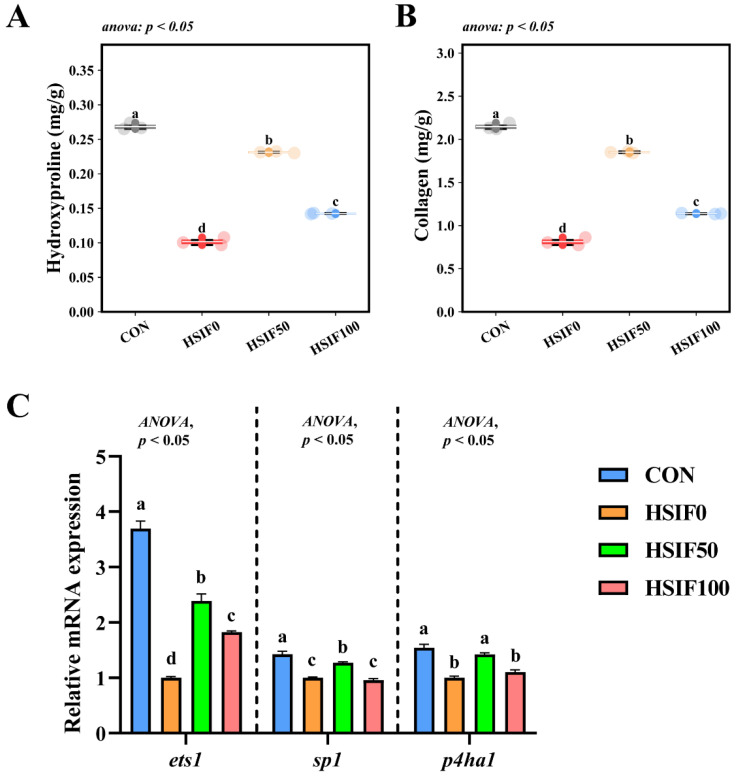
Effects of dietary treatments on muscle collagen content and expression of collagen metabolism-related genes in *M. albus* (*n* = 4). Notes: (**A**) hydroxyproline content of muscle; (**B**) collagen content of muscle; (**C**) ets1—ETS proto-oncogene 1; sp1—Sp1 transcription factor; p4ha1—Prolyl 4-hydroxylase subunit alpha 1. Bars represent mean ± standard error (SE). Different superscript letters denote significant differences among groups (*p* < 0.05). CON: control diet (6.16% crude fat); HSIF0: high-fat diet (11.98% crude fat); HSIF50: HSIF0 supplemented with 50 mg/kg soy isoflavones; HSIF100: HSIF0 supplemented with 100 mg/kg soy isoflavones.

As shown in Figure 5A,B, muscle hydroxyproline and collagen contents were significantly lower in the HSIF0 group than in the CON and soy isoflavone-supplemented groups (*p* < 0.05). Furthermore, the HSIF100 group exhibited significantly lower hydroxyproline and collagen levels compared with the HSIF50 group (*p* < 0.05).

As shown in Figure 5C, the relative expression levels of ets1, sp1, and p4ha1 in muscle were significantly downregulated in the HSIF0 group compared with the CON group (*p* < 0.05). Supplementation of the high-fat diet with 50 mg/kg soy isoflavones significantly upregulated these genes compared with the HSIF0 group (*p* < 0.05). However, as the soy isoflavone supplementation level increased, the relative expression of ets1, sp1, and p4ha1 declined significantly (*p* < 0.05).

### 3.5. Antioxidant Capacity

**Figure 6:**
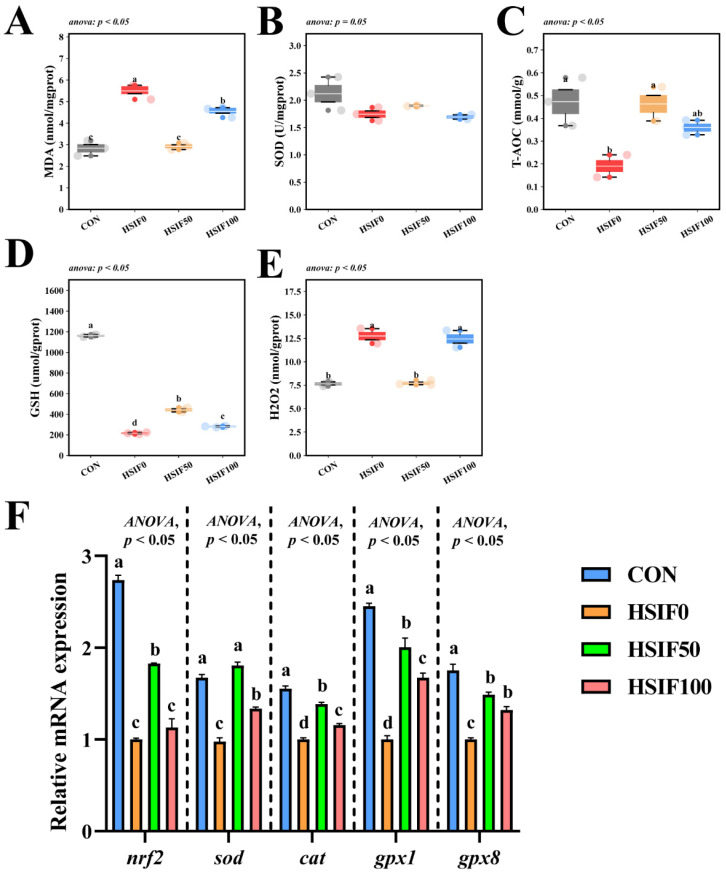
Effects of dietary treatments on antioxidant capacity in *M. albus* (*n* = 4). Notes: (**A**–**E**) represent reactive malondialdehyde, superoxide dismutase, total antioxidant capacity, glutathione, and hydrogen peroxide, respectively. (**F**) nrf2—Nuclear factor erythroid 2-related factor 2; sod—Superoxide dismutase; cat—Catalase; gpx1—Glutathione peroxidase 1; gpx8—Glutathione peroxidase 8. Bars represent mean ± standard error (SE). Different superscript letters denote significant differences among groups (*p* < 0.05). CON: control diet (6.16% crude fat); HSIF0: high-fat diet (11.98% crude fat); HSIF50: HSIF0 supplemented with 50 mg/kg soy isoflavones; HSIF100: HSIF0 supplemented with 100 mg/kg soy isoflavones.

As shown in Figure 6A–E, muscle SOD activity did not differ significantly among treatments (*p* > 0.05). Compared with the CON group, the HSIF0 group exhibited significantly higher MDA and H_2_O_2_ contents, but significantly lower T-AOC activity and GSH content (*p* < 0.05). Compared with the HSIF0 group, supplementation of the high-fat diet with 50 mg/kg soy isoflavones significantly reduced MDA and H_2_O_2_ contents, while significantly increasing T-AOC activity and GSH content (*p* < 0.05). Furthermore, the HSIF100 group showed significantly higher MDA and H_2_O_2_ contents and significantly lower GSH content than the HSIF50 group (*p* < 0.05).

As shown in Figure 6F, compared with the CON group, the HSIF0 group displayed significantly lower relative mRNA expression levels of nrf2, sod, cat, gpx1, and gpx8 (*p* < 0.05). Compared with the HSIF0 group, dietary supplementation with 50 mg/kg soy isoflavones significantly upregulated the expression of these genes (*p* < 0.05). In addition, as the level of soy isoflavone supplementation increased, the expression levels of nrf2, sod, cat, and gpx1 were significantly downregulated (*p* < 0.05).

## 4. Discussion

### 4.1. Proximate Composition, Muscle Oil Red O-Stained Sections, and Fatty Acid Profile

In the present study, feeding rice field eels a high-fat diet (HSIF0) significantly elevated crude lipid contents in whole-body and muscle tissues compared to the control group (CON). Additionally, Oil red O-staining revealed extensive lipid droplet accumulation along myosepta and between muscle fibers in the HSIF0 group, indicative of abnormal lipid deposition under high-fat feeding conditions. This phenomenon aligns with previous findings in various fish species, such as mirror carp (*Cyprinus carpio*) [21] and yellow catfish [6], where high-fat diets promote excessive lipid accumulation in tissues, potentially impairing overall health. In contrast, supplementation with soy isoflavones effectively reduced these elevated lipid levels. This hypolipidemic effect of soy isoflavones has been well-documented in mammals and livestock, where they modulate lipid metabolism by suppressing lipogenesis, enhancing fatty acid oxidation, and improving blood lipid profiles [22]. In rodents, soy isoflavones have been shown to decrease body weight, hepatic lipid accumulation, and serum total and LDL cholesterol through mechanisms involving peroxisome proliferator-activated receptors (PPARs) and reduced intestinal cholesterol absorption [23]. Extending to aquatic species, soy isoflavones exhibit similar lipid-lowering properties in fish. In juvenile Chinese mitten crab, dietary soy isoflavones improved growth while inhibiting lipid synthesis genes and promoting decomposition, thereby reducing fat accumulation in high-fat diets [16]. Additionally, in common carp, genistein (a key soy isoflavone) modulated lipid metabolism, antioxidant activity, and immunity, alleviating high-fat-induced lipid buildup [24]. These findings suggest that soy isoflavones may mitigate high-fat-induced lipid dysregulation in rice field eels through comparable pathways, potentially involving antioxidant enhancement and gene regulation of lipid metabolism.

Regarding fatty acid composition, the high-fat diet altered muscle profiles by decreasing ΣSFA, ΣMUFA, and Σn-3/Σn-6 ratios while increasing Σn-6 across all high-fat groups compared to CON, reflecting a shift toward pro-inflammatory profiles often observed in fish under lipid overload. This is corroborated by studies in spotted seabass (*Lateolabrax maculatus*), where high-fat feeding disrupts n-3/n-6 balance, elevates n-6 PUFAs, and reduces beneficial ratios, impacting growth and tissue quality [25]. However, soy isoflavone supplementation (HSIF50 and HSIF100 groups) elevated ΣPUFA and Σn-3 relative to CON, and in HSIF50 specifically, lowered ΣSFA and Σn-6 while boosting ΣMUFA and EPA+DHA compared to HSIF0. This indicates that soy isoflavones can favorably modulate fatty acid profiles, potentially by enhancing desaturase activity or reducing n-6 dominance [26]. Similar effects have been reported in grass carp, where soy isoflavones combined with oils increased n-3 PUFA deposition, including EPA and DHA, in muscle tissues [27]. The observed increases in EPA+DHA likely contribute to improved muscle quality and antioxidant performance in soy isoflavone-supplemented groups; this will be proven in the indicators to be tested next. Taken together, these results indicate that soy isoflavones alleviate high-fat-induced lipid dysregulation and improve fatty-acid composition, laying the foundation for enhanced muscle quality.

### 4.2. Muscle Water-Holding Capacity and Textural Properties

The high-fat diet significantly impaired muscle water-holding capacity in rice field eels, as evidenced by increased cooking loss, liquid loss, and water loss compared to the control group. This deterioration is likely attributable to excessive lipid accumulation in muscle tissues, which disrupts myofibrillar protein structures, reduces the space for water retention, and promotes drip loss during processing and storage [28]. Similarly, the HSIF0 group exhibited diminished textural properties, including lower hardness, cohesiveness, gumminess, chewiness, and resilience, reflecting a softer and less elastic muscle matrix, potentially due to fat infiltration altering collagen and protein integrity [29]. These findings are consistent with observations in other aquatic species under high-fat feeding regimes. For example, in Nile tilapia, high-fat diets compromised water-holding capacity and led to looser flesh texture by inducing lipid overload and oxidative damage [3]. Comparable effects have been reported in grass carp, where elevated dietary lipids decreased muscle pH and water-holding capacity, correlating with reduced firmness and increased tenderness [30].

Supplementation with soy isoflavones at 50 mg/kg effectively ameliorated these impairments, significantly lowering cooking loss, liquid loss, and water loss while enhancing hardness, cohesiveness, gumminess, chewiness, and resilience relative to the HSIF0 group. However, higher supplementation at 100 mg/kg resulted in elevated liquid and water losses and reduced cohesiveness, gumminess, and chewiness compared to HSIF50, possibly through over-modulation of metabolic pathways. The hypolipidemic and muscle quality-enhancing properties of soy isoflavones have been extensively documented in mammals and livestock, where they mitigate lipid peroxidation, stabilize protein matrices, and improve water retention and texture by activating antioxidant defenses and reducing fat deposition. In broilers, dietary soy isoflavones enhanced growth performance, meat quality, and water-holding capacity while decreasing oxidative stress and lipid accumulation [10]. Extending to aquatic species, soy isoflavones demonstrate analogous protective effects against high-fat-induced muscle degradation. In Chinese mitten crabs, supplementation improved lipid metabolism and flesh quality under high-fat conditions, enhancing texture and reducing losses associated with water-holding [31]. In rainbow trout, diets incorporating soy-derived isoflavones maintained muscle integrity and quality traits, countering potential anti-nutritional factors while supporting better textural properties [31]. Collectively, these findings demonstrate that moderate SIF supplementation improves water-holding capacity and textural attributes in rice field eel muscle.

### 4.3. Muscle Histomorphology and Expression of Myogenesis-Related Genes

To further elucidate how soy isoflavones improved water-holding capacity and texture under high-fat feeding, we assessed muscle microstructure and the expression of myogenesis-related regulators. The high-fat diet induced notable histopathological alterations in rice field eel muscle, as evidenced by HE-stained sections showing loosely arranged fibers, irregular morphology, enlarged inter-fiber spaces, and partial structural disruption. Concurrently, it upregulated the expression of mstn while downregulating myod, tcap, mrf4, and mrf5, indicating impaired myogenic processes under lipid overload. These changes align with observations in other fish species, like the yellow catfish [6] and grass carp [4], where high-fat diets compromise muscle histology, fiber quality, and gene regulation. These demonstrated that excessive dietary fats promote lipotoxicity, oxidative damage, and hinder muscle development [28].

Supplementation with 50 mg/kg soy isoflavones effectively ameliorated these issues, restoring compact fiber arrangement, uniform morphology, and reduced inter-fiber spaces, while downregulating mstn and upregulating tcap, mrf4, and mrf5 to enhance myogenic activity. However, at the HSIF100 group, the inter-fiber gaps that widened were less pronounced, and gene expression partially reverted, suggesting a dose-dependent optimum beyond which benefits diminish. In aquatic species, soy isoflavones exhibit similar benefits in enhancing muscle quality and histology. In juvenile Chinese mitten crab, soy isoflavones improved growth, antioxidant capacity, and lipid metabolism, which could indirectly support better muscle structure under high-fat conditions [16]. In rainbow trout, dietary soy isoflavones influenced tissue deposition and fillet quality, maintaining health without adverse effects at appropriate levels [32]. These findings imply that soy isoflavones in rice field eels likely act through antioxidant enhancement, lipid modulation, and estrogenic mimicry to optimize histological architecture and myogenic gene networks at moderate doses. The suboptimal effects at higher doses may stem from excessive isoflavone exposure, inducing stress or endocrine disruptions, as high doses have been linked to increased oxidation in tissues and potential toxicity in animals [7]. In fish, elevated soy isoflavones can alter endocrine functions and induce stress responses, such as changes in cortisol levels, which might counteract protective benefits [17]. Furthermore, these histopathological improvements and gene expression shifts in the HSIF50 group further corroborate the enhanced water-holding capacity and textural properties, while the partial histological and genetic reversals at higher doses aligned with declines in cohesiveness and chewiness. Overall, these results suggest that 50 mg/kg SIFs restore muscle fiber organization and activate myogenic pathways, thereby supporting muscle structure and function under high-fat feeding, whereas higher doses diminish these benefits.

### 4.4. Muscle Collagen Content and Expression of Collagen Metabolism-Related Genes

In grass carp, dietary manipulations affecting protein and lipid balance altered muscle hardness through changes in collagen metabolism, with reduced levels correlating to softer textures [33]. Similarly, in zebrafish (*Danio rerio*), high-fat feeding induced muscle mitochondrial dysfunction and impaired fiber characteristics, indirectly impacting collagen integrity via oxidative stress and metabolic shifts [34]. This aligns with findings in this study, the high-fat diet significantly reduced muscle hydroxyproline and collagen contents while downregulating the expression of collagen metabolism-related genes ets1, sp1, and p4ha1 compared to the control group, indicating impaired collagen synthesis and structural integrity under lipid overload.

The HSIF50 group effectively reversed these deficits, elevating hydroxyproline and collagen levels while upregulating ets1, sp1, and p4ha1 to enhance collagen metabolism. However, at the HSIF100 group, these parameters declined relative to HSIF50, suggesting an optimal dose threshold. The collagen-promoting properties of soy isoflavones are well-documented in mammals and livestock, where they support skeletal health by modulating gene expression and extracellular matrix formation. In orchidectomized rats, soy isoflavones elevated mRNA levels of type I collagen and osteocalcin, demonstrating anabolic influences on connective tissues [35]. In Chinese mitten crabs, soy isoflavones optimized gonadal and muscle parameters, including enhanced collagen content for better flavor and texture [36]. In grass carp, dietary soy isoflavones increased water-holding capacity and physical properties, largely attributed to boosted collagen deposition [27]. These results imply that soy isoflavones in rice field eels may facilitate collagen anabolism through transcriptional activation support at moderate doses. Furthermore, the elevated collagen content in the HSIF50 group further substantiates the improved textural properties and water-holding capacity, such as increased hardness and chewiness, alongside reduced cooking, liquid, and water losses, as collagen networks enhance muscle matrix stability and moisture retention. In summary, moderate SIF supplementation enhanced collagen deposition and metabolism, reinforcing muscle integrity and textural properties, whereas higher doses were less effective, pointing to a dose-dependent optimum.

### 4.5. Antioxidant Capacity

In aquaculture species, muscle antioxidant capacity is strongly diet-dependent, and the redox status in turn exerts significant regulatory effects on lipid metabolism and the resulting fatty-acid composition [37]. The high-fat diet (HSIF0) significantly compromised muscle antioxidant capacity in rice field eels, as indicated by elevated MDA and H_2_O_2_ contents, reduced T-AOC activity and GSH levels, and downregulated expression of nrf2, sod, cat, gpx1, and gpx8 genes, reflecting heightened oxidative stress and diminished defense mechanisms under lipid overload. This pattern accords with reports in multiple fish species: high-fat feeding drives lipid peroxidation and ROS accumulation, impairs antioxidant enzymes, and consequently aggravates inflammation and metabolic dysregulation [38,39,40].

At 50 mg/kg, soy isoflavones lowered MDA and H_2_O_2_, elevated T-AOC and GSH, and upregulated nrf2, sod, cat, gpx1, and gpx8, indicating strengthened antioxidant defenses. By contrast, 100 mg/kg partially reversed these gains, suggesting a dose-dependent optimum. Similar enhancements of antioxidant status and immune function have been reported in turbot and juvenile golden pompano under stress challenges—dietary soy isoflavones improved antioxidant status and immune responses [11,12]. Collectively, these findings suggest that moderate isoflavone dosing activates Nrf2-mediated cytoprotection and complements its hypolipidemic effects, whereas higher dosing may cross a hormetic threshold and exhibit pro-oxidant or tolerance effects. The improved redox capacity at HSIF50 also aligns with the higher muscle EPA+DHA, helping to augment antioxidant defenses and mitigate lipid peroxidation, contributing to overall muscle quality improvement. Overall, SIF at a dosage of 50 mg/kg improved muscle quality through lipid regulation, collagen synthesis, and activation of antioxidants, while 100 mg/kg resulted in decreased yields.

### 4.6. Economic and Regulatory Considerations

In addition to the biological benefits observed, the potential economic and regulatory aspects of soy isoflavone application in aquafeeds should also be considered. From an economic perspective, the cost of incorporating soy isoflavones must be weighed against the improvements in fish growth, flesh quality, and overall health. In practice, only about 50 g of soy isoflavones is required per metric ton of feed to achieve the optimal effect observed in this study, corresponding to an additional cost of approximately 20 RMB per ton, which is generally acceptable for commercial aquaculture operations. From a regulatory standpoint, soy isoflavones are naturally derived compounds that are generally recognized as safe and have already been approved for use in livestock and poultry feeds. However, their application in aquafeeds is less established, and determining appropriate supplementation gradients will be crucial for ensuring both safety and efficacy in aquatic species. Therefore, further studies on long-term safety, optimal inclusion levels, and regulatory compliance will be essential to facilitate the practical adoption of soy isoflavone supplementation in aquaculture.

## 5. Conclusions

This study demonstrated that dietary soy isoflavone supplementation can effectively mitigate the adverse effects of a high-fat diet on muscle quality, collagen deposition, and antioxidant capacity in *M. albus*. Specifically, supplementation at 50 mg/kg reduced muscle lipid accumulation, improved water-holding capacity and textural properties, promoted collagen synthesis, and enhanced antioxidant defense by modulating the expression of myogenesis-, collagen metabolism-, and antioxidant-related genes. However, excessive supplementation at 100 mg/kg partially attenuated these beneficial effects, indicating a dose-dependent response. Collectively, our findings suggest that 50 mg/kg is the optimal inclusion level of soy isoflavones in high-fat diets for *M. albus*, providing practical guidance for aquafeed formulation strategies aimed at improving flesh quality and health status in aquaculture. Beyond aquaculture, these findings may offer insights into vertebrate muscle biology and nutrition; however, extrapolation to humans requires dedicated studies. Limitations of this study include its focus on a single species, short-term feeding duration, and the lack of protein-level or functional validation of signaling pathways. Future research should, therefore, involve cross-species validation, long-term feeding trials, and confirmatory studies of the molecular mechanisms underlying the observed effects.

## Figures and Tables

**Table 1:** Feed formula and nutrition levels (dry matter).

Ingredient	CON	HSIF0	HSIF50	HSIF100
Fish meal	40.00	40.00	40.00	40.00
Soy protein concentrate	16.00	16.00	16.00	16.00
Poultry by-product meal	3.00	3.00	3.00	3.00
Beer yeast	5.00	5.00	5.00	5.00
Fish oil	2.20	8.20	8.20	8.20
Microcrystalline cellulose	10.26	4.26	4.26	4.26
Corn starch	20.00	20.00	20.00	20.00
Choline	0.50	0.50	0.50	0.50
Ca(H_2_PO_4_)_2_	2.00	2.00	2.00	2.00
Premix ^1^	1.00	1.00	1.00	1.00
Antioxidants ^2^	0.01	0.01	0.01	0.01
Mold inhibitor ^3^	0.03	0.03	0.03	0.03
Soy isoflavone ^4^, mg/kg	0.00	0.00	50.00	100.00
Nutrition levels ^5^				
Moisture	7.45	7.52	7.56	7.49
Crude protein	41.36	41.26	40.96	41.51
Crude fat	6.16	11.98	12.11	12.08
Ash	10.23	10.35	10.17	10.31

Notes: ^1^ Provided by MGO Ter Bio-Tech Co., Ltd. (Qingdao, China). Composition consistent with previous studies [2]. ^2^ The main component of antioxidant is ethoxyquin. ^3^ The main component of mould inhibitor is calcium propionate. ^4^ ≥98%, purchased from Chengdu Yuancheng Technology Co., Ltd. (Chengdu, China). ^5^ The crude protein, crude lipid, and ash levels were measured values.

**Table 2:** Primes of hepatic glycolipid metabolism, antioxidant and inflammatory related genes of *M. albus*.

Gene	Forward Primer (5′-3′)	Reverse Primer (5′-3′)	Accession No.
*rpl17*	CGAGAACCCGACTAAATCA	GTTGTAGCGACGGAAAGG	XM_020587712.1
*mstn*	TTGGCTGGGACTGGATTATTG	TTGGTGACATCTTGGTGGGG	KM103284.1
*myod*	ACATCCAGCTCCTTTCCTCC	TGCTCAATCCCAAACACACC	XM_020593504.1
*tcap*	TGGAGAAGTGTAATGGTATGGTGG	GCCTTTCCTGAGGTTGGGTT	XM_020607417.1
*mrf4*	CAAGGTGGAGATTTTACGCAGC	GTTTGGGTTTTCTCCTGTTCGT	KM103283.1
*myf5*	ATGTCTTCTCATCATCCCAGGTC	CCTGACGTGCTCATCTTCCTCT	KM103285.1
*ets1*	TCAGCTCAGAGGAACTGCTGAC	AGTCCTGGCCACCAAGTTTACC	XM_020600359.1
*sp1*	ACAGCCAGTGTCTTCCAACAG	AAGGATCTGCTGTGACTGCTG	XM_020600700.1
*p4ha3*	AAAGACTCTGCCAGACCCAAG	ATGTCTTCAGCCTCTGTGTCAG	XM_020588625.1
*nrf2*	TCACAGACGAGAATGATGCC	CTGCTACTGGGAACTGAAACTG	XM_020596408.1
*sod*	GTTGCCAAGATAGACATCACGG	TCATTGCCTCCTTTTCCCAG	XM_020598412.1
*cat*	CATTGGGAAGACTACACCTATCGC	GATGAAGAAGATGGGGGTGTTG	XM_020624985.1
*gpx1*	AGATGTGAATGGGAAGGATGCC	AAACTTCGGGTCAGTCATCAGG	XM_020607739.1
*gpx8*	ATCCTGCCTTCAGATTCCTCAC	TCATTTCTCGCACCAGCACT	XM_020593975.1

Notes: RPL17 was used as the reference gene.

## Data Availability

The datasets used and/or analyzed in the current study are available from the corresponding author on reasonable request.

## Supplementary Materials

The following supporting information can be downloaded at: https://www.mdpi.com/article/10.3390/antiox14101195/s1, Figure S1: Schematic representation of the experimental design.
