# Supplementary material for: Dietary Soy Isoflavones Ameliorate Muscle Quality in High-Fat Diet-Fed Rice Field Eels (Monopterus albus) by Modulating Myogenesis, Collagen Synthesis, and Antioxidant Capacity
Source: Antioxidants (Basel). 2025 Oct 1;14(10):1195. doi: 10.3390/antiox14101195 (PMC12561997; doi:10.3390/antiox14101195)
Supplement: Supplementary file 1 [file antioxidants-14-01195-s001.zip › antioxidants-3907844-supplementary.pdf]

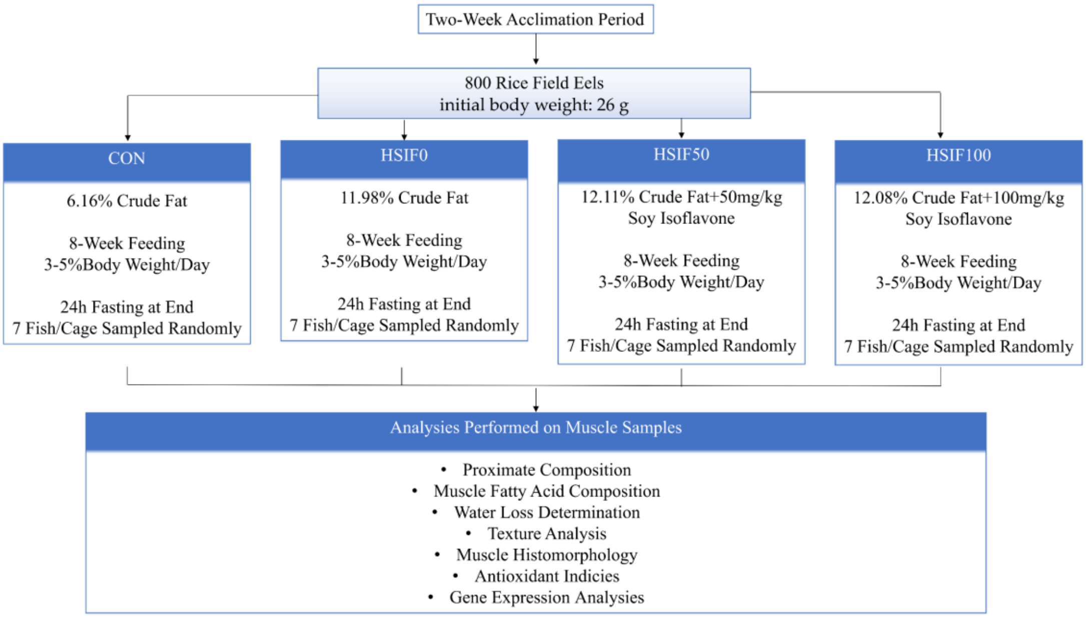

Figure S1. Schematic representation of the experimental design. After a two-week acclimation period, 800 rice field eels (*Monopterus albus*, initial body weight: 26 g) were randomly assigned to four dietary treatments: CON (control diet, 6.16% crude fat), HSIF0 (high-fat diet, 11.98% crude fat), HSIF50 (high-fat diet + 50 mg/kg soy isoflavones, 12.11% crude fat), and HSIF100 (high-fat diet + 100 mg/kg soy isoflavones, 12.08% crude fat). Fish were fed once daily at 3–5% of body weight for eight weeks. At the end of the trial, following 24 h fasting, seven fish were randomly sampled from each cage. Muscle samples were collected for proximate composition, fatty acid composition, water loss determination, texture analysis, histological observation, antioxidant indices, and gene expression analyses.
